# The Prevalence, Risk Factors and Clinical Correlates of QTc Prolongation in Chinese Hospitalized Patients With Chronic Schizophrenia

**DOI:** 10.3389/fpsyt.2021.704045

**Published:** 2021-08-16

**Authors:** Haiyan Cao, Yongjie Zhou, Tao Li, Cong Yao, Weiliang Yang, Siying Kong, Yanyan Wang, Baoping Yu, Qingyan Jiao, Yun Sun, Xiaoju Jia, Yuting Wang, Zhonggang Wang, Xiangyang Zhang, Jie Li

**Affiliations:** ^1^Institute of Mental Health, Tianjin Anding Hospital, Tianjin Medical University, Tianjin, China; ^2^Department of Psychiatric Rehabilitation, Shenzhen Kangning Hospital, Shenzhen, China; ^3^Department of Psychiatry, Jining Psychiatric Hospital, Jining, China; ^4^Key Laboratory of Mental Health, Institute of Psychology, Chinese Academy of Sciences, Beijing, China

**Keywords:** schizophrenia, antipsychotics, QTc prolongation, Chinese, prevalence, risk factor, clinical correlates

## Abstract

**Background:** The QTc interval may be significantly prolonged in schizophrenia patients taking antipsychotics. Few studies have addressed QTc prolongation (QTP) in Chinese patients.

**Objectives:** This study was designed to evaluate the prevalence of QTP and its clinical correlates in Chinese hospitalized patients with chronic schizophrenia.

**Methods:** A total of 436 inpatients and 291 normal controls matched with age and sex were included. QTc prolongation was defined as 2 standard deviations (SD) above the mean value of normal controls. Positive and Negative Syndrome Scale (PANSS) and its five-factor model were used to evaluate psychopathological symptoms.

**Results:** QTc interval was significantly longer in patients than in normal controls. The prevalence of QTP is 8.26% in Chinese hospitalized patients with chronic schizophrenia. More women than men displayed QTP. Compared with patients without QTP, the patients with QTP had significantly higher concrete/disorganized subscore, lower low density lipoprotein (LDL) and lower total protein (TP). Furthermore, binary logistic regression analysis showed that higher number of hospitalizations, higher concrete/disorganized subscore and lower LDL were risk factors for QTP. Correlation analysis indicated significant association between QTc interval and the following variables: sex, age, duration of illness, the number of hospitalizations, PANSS total score, fasting blood glucose (FPG). Finally, a multiple regression analysis showed that older age, antipsychotic polypharmacy, higher PANSS total score, and lower LDL were risk factors for QTP. Among them, LDL seemed to be a protective factor for QTP.

**Conclusions:** QTc interval was longer in schizophrenia patients than in normal controls. The prevalence of QTP is 8.26% in Chinese hospitalized patients with chronic schizophrenia. Some clinical characteristics were risk factors for QTP. And LDL seemed to be a protective factor for QTP.

## Introduction

Schizophrenia is a chronic, serious and complex psychiatric disorder that occurs worldwide. The estimated life expectancy of schizophrenia patients is reduced by 10–20 years (1). Schizophrenia patients have significantly higher mortality rates than the general population and the risk of dying from cardiovascular disease is especially increased (2). This may be related to the increased risk of poor diet, sedentary lifestyle, dyslipidemia, weight gain, obesity, type 2 diabetes and arrhythmia in schizophrenia patients, some of which may be associated with antipsychotics (3). Specifically, exposure to antipsychotics conveys a more than 2-fold risk of sudden cardiac death (SCD), which is thought to be mediated by effects on QT interval prolongation and the risk of torsade de pointes (TdP) (4). As we all know, antipsychotics could cause QT interval prolongation (5). Prolonged QT interval is a high risk factor for ventricular tachycardia and torsade de pointes (TdP), which could eventually deteriorate to fatal ventricular fibrillation and even lead to sudden cardiac death (SCD) if it not be treated immediately (6).

QT interval starts with the beginning of QRS complex and ends with the termination of T wave. QT interval represents the time of ventricular depolarization and repolarization and is usually used the formula of Bazett (QTc = QT/RR^0.5^) to correct heart rate, as the heart rate has an effect on the duration of the QT interval (7). Normally, the average QTc interval is about 410–430 ms for adult males and 420–430 ms for adult females (8). QTc intervals >500 ms are associated with a 2-to 3-fold increase in TdP risk. This threshold has been recommended for drug discontinuation both for men and women (9).

At present, QTc prolongation (QTP) in patients with schizophrenia has been widely concerned. Moreover, when prescribing psychotropic drugs we should pay more attention to the prolongation of QTc interval (10). With respect to the prevalence of QTP, the results of different studies vary greatly, ranging from 0.5 to 38% (11, 12). There are few reports on the prevalence of QTP in Chinese schizophrenia patients.

There are many factors that could affect QTP, including age, gender, comorbid cardiovascular disease, electrolyte disturbance, types of antipsychotics and genetic susceptibility, etc. (4, 13, 14). Female gender and old age are well-known risk factors (15, 16). In addition, some studies have found that age and sex were not associated with QTc prolongation (17). Comorbid cardiovascular disease is also a risk factor for QTP, such as coronary heart disease, arrhythmia (11).

Different antipsychotics have different effects on QTP. Among second-generation antipsychotics, some of them could cause QTc prolongation, such as quetiapine, olanzapine, risperidone, ziprasidone and so on (18). There are also studies found that olanzapine does not prolong QTc interval (19). Some studies suggested that aripiprazole was not associated with significant QTc prolongation (18), yet other studies observed that aripiprazole could decrease the risk of QTc prolongation (11, 20). In the first-generation antipsychotics, haloperidol is associated with significant QTc prolongation (18). Many researchers found that antipsychotic polypharmacy also increases the risk of QTc interval prolongation, which is considered dose-dependent (11, 21). Moreover, a systematic review found that the available evidence fails to prove that antipsychotic polypharmacy generally worsens the QTc prolongation (22). A Japanese study on 1,543 schizophrenia patients also showed that two antipsychotics did not increase the risk of QTc interval prolongation (23). Therefore, the results of some studies in this area are inconsistent.

So far it is still unclear whether some factors are associated with QTP. These studies that identified factors associated with QTP are mainly of Caucasians, and some results are inconsistent. There are few studies on Asian population, and even less on Chinese population. There are only three studies on Chinese population, one of them studied the gender difference and clinical correlates of QTc prolongation in Chinese chronic hospitalized patients with schizophrenia (24), another one was a study on the prolongation of QTc interval in the first-episode schizophrenia patients after short-term antipsychotic treatment (25), and the other study mainly studied the effects of different antipsychotics on QTP and related genes of QTP in the first-episode schizophrenia patients (26).

Compared with the first-episode schizophrenia patients, patients with chronic schizophrenia have increased risk of poor diet, sedentary lifestyle, dyslipidemia, weight gain, obesity, type 2 diabetes, comorbid other diseases, concomitant medications. This situation increases the potential risk factors that may prolong QTc interval in patients with chronic schizophrenia. It is still unclear whether some factors could affect QTP, and there are still some factors that have not been included in previous studies, such as the duration of illness, the number of hospitalizations, family history of mental illness, severity of schizophrenia and so on.

Furthermore, there were few studies addressed the prevalence of QTP in Chinese schizophrenia patients. The objectives of this naturalistic study are to evaluate the prevalence of QTP in a relatively large population of Chinese hospitalized patients with chronic schizophrenia, and to explore its relationship with clinical characteristics, metabolic parameters and antipsychotic treatment. Moreover, compared with previous studies, we have included as many possible related factors in this study as possible.

## Materials and Methods

### Subjects

This was a naturalistic cross-sectional study. A total of 436 schizophrenia patients hospitalized in Tianjin Anding Hospital were included in this study, all of whom were unrelated Han Chinese. The inclusion criteria were as follows: (1) The patients were diagnosed with schizophrenia independently by two psychiatrists using the Chinese version of Structured Clinical Interview for DSM-5 (SCID). (2)The patients were 18–70 years old, regardless of gender. (3) The duration of illness was more than 2 years. (4) Patients were treated with stable oral doses of antipsychotics for at least 6 months. (5) Patients and their families agreed to participate in this study and signed informed consent.

Patients with severe somatic diseases (such as cardiovascular, liver, kidney, gastrointestinal diseases, etc.), infectious diseases and immune system diseases, severe neurological diseases and intellectual disability, pregnancy and lactation, substance abuse were excluded from this study. In addition, other factors that may affect QT interval will also be excluded, such as arrhythmia, family history of prolonged QT interval, abnormal thyroid function, electrolyte disorder such as hypokalemia, etc.

All patients enrolled in the study were asked to complete a questionnaire to collect general information, demographic characteristics, smoking status, and medical conditions. The questionnaire survey was conducted by trained research staff. For all patients, a detailed medical history, physical examination, laboratory examination were obtained. None of the 436 patients we enrolled in this study met the criteria for alcohol or drug abuse or dependence.

All patients in this study were hospitalized patients in the same hospital. From admission, the patients' diet and daily life were uniformly managed by the hospital. Three meals a day with a balanced diet are provided by the hospital. Patients also had the opportunity to exercise for an hour at a fixed time every day. Occasionally, their family members or friends brought some fruits or snacks as supplementation when visiting. Therefore, the patients in this study could be regarded as a representative sample of hospitalized patients with chronic schizophrenia in China.

A total of 291 normal controls (mean age, 44.72 ± 9.03 years; male/female = 203/88) were recruited from local communities in Tianjin, and matched for sex and age. We used this normal control group to establish the upper limits of the normal values for measurement of QTc interval. The inclusion criteria of the normal control group were no psychiatric disorders, cardiovascular diseases, severe somatic diseases, pregnancy and lactation. The standardized 12-lead resting electrocardiograms (ECGs) were obtained from all the normal controls. Any abnormalities found in the ECG will be ruled out, even if there is no previous history of cardiovascular disease. Psychiatric evaluations were conducted by two psychiatrists to rule out psychiatric disorders among controls. Their current mental state, personal history and family history of mental disorders were evaluated by two research psychiatrists through unstructured clinical interviews. None of them displayed a family history of psychiatric disorder.

After a psychiatrist introduced the research protocol to the potential subjects in a language appropriate to the comprehension level of the subjects, the subjects signed informed consent document. The investigation was carried out in accordance with the latest version of the Declaration of Helsinki. Before this study began, the Human Ethics Committee of Tianjin Anding Hospital approved our research protocol.

### Measurement of QTc

The standardized 12-lead resting electrocardiograms (ECGs) at 25 mm/s were obtained from all the subjects between 8:00 and 12:00 a.m., using the same electrocardiograph with automatic analysis function. When obtaining the ECG, before recording the ECG the patient's heart rate was stable for 1–2 min. QT interval was measured from the beginning of QRS complex to the end of T wave. To correct the effect of heart rate on QT interval, we calculated QTc interval according to the Bazetts formula (QTc = QT/RR^0.5^). The ECG information we recorded, including heart rate, QT interval, QTc interval and ECG diagnosis, has been confirmed by cardiologists. The electrocardiograph used in the study was CM100 produced by China Shenzhen COMEN Medical Equipment Co., Ltd.

### Clinical Assessment

In this study, we used Positive and Negative Syndrome Scale (PANSS) to assess the psychopathology of the subjects. PANSS was evaluated by two psychiatrists who had extensive clinical experience and received training on how to rate PANSS simultaneously. In the course of the study, the repeated assessments of PANSS total score led to a correlation coefficient of >0.8 between the two scorers. The composition of original PANSS included seven items of positive subscale, seven items of negative subscale and 16 items of general psychopathology subscale (27). Later, Wallwork et al. proposed the five-factor model which was considered to better explain the structure of PANSS (28, 29). The five-factor model was composed of the following five factors: a positive factor included P1, P3, P5, and G9; a negative factor included N1, N2, N3, N4, N6, and G7; a concrete/disorganized factor included P2, N5, and G11; an excited factor included P4, P7, G8, and G14; a depressed factor included G2, G3, and G6.

### Assessments of Anthropometric Variables

Height and body weight were measured in a standardized fashion. Body mass index (BMI) was calculated according to the formula: BMI = weight in kilogram/square of height in meters. Height was measured to millimeters with the subjects standing upright and barefooted. Body weight was evaluated with an electronic scale (EB9005L, Xiangshan, China) with light indoor clothing calibrated to 0.1 kg.

### Measurements of Blood Sample

Venous blood samples were collected from all patients between 7 and 9 a.m., after fasting for at least 8 h. Then the plasma samples were sent to the diagnostic laboratory of Tianjin Anding hospital immediately for testing. All the hematological indicators, including fasting blood glucose (FPG), total cholesterol (CHOL), triglyceride (TG), high density lipoprotein (HDL) cholesterol, low density lipoprotein (LDL) cholesterol, total protein (TP) and albumin (ALB), were measured in the same diagnostic laboratory.

### Statistical Analysis

Categorical variables were expressed as percentages, and the continuous variables were expressed by means and standard deviations (x ± s). Most of the demographic data, clinical data, clinical assessment, the QTc intervals and the hematological indicators were normally distributed in patients except for the number of hospitalizations, chlorpromazine equivalent dose and plasma lipid profile variables (Kolmogorov-Smirnov one-sample test). Then log transformation was used to normalized these variables that did not conform to the normal distribution. All patients were divided into two groups according to whether the QTc interval was prolonged or not. We compared the categorical variables between the two groups by using chi-square test. The comparison of normally distributed data between the two groups was analyzed by using the *t*-test for continuous variables of two independent samples. And the comparison of data that did not fit the normal distribution was analyzed by the non-parametric test of two independent samples. Moreover, analysis of covariance (ANCOVA) was performed to control for the effects of age and sex on QTc interval prolongation and metabolic disturbances. Furthermore, a binary logistic regression analysis was conducted to evaluate which factors were significantly associated with QTc prolongation. Pearson or Spearman correlation coefficients were further performed to examine the interrelation among QTc interval and demographic data, clinical variables and hematological indicators. Finally, a multiple regression analysis was utilized to identify the significant predictive variables associated with QTc interval, including the covariates of sex, age, duration of illness, the number of hospitalizations, family history of mental illness, antipsychotic polypharmacy, antipsychotic dose (mg/day) (chlorpromazine equivalents), smoking behavior, PANSS total score, FPG, LDL, and ALB. In addition, Bonferroni corrections were performed to adjust for multiple tests. SPSS 23.0 was used to complete all statistical analysis. All *p*-values were 2 tailed, and the significance level was set at 0.05.

## Results

### The Demographics and ECG Datas of Patients and Healthy Controls

The demographics and ECG datas of schizophrenia patients and healthy controls are summarized in Table 1.The average age of schizophrenia patients was 45.99 ± 12.52 years. The mean age of healthy controls was 44.72 ± 9.03 years. And there was no difference in age and gender between patients and healthy controls (both *p* > 0.05). The QTc interval of patients was longer than that of healthy controls, and the difference was statistically significant (*t* = 7.071, *p* = 0.000). In addition, compared with male healthy controls, the QTc interval of male patients was longer, and the difference was statistically significant (*t* = 5.647, *p* = 0.000). Similarly, female patients also had a longer QTc interval compared with female healthy controls, and the difference was statistically significant (*t* = 4.117, *p* = 0.000).

**Table 1:** The demographics and ECG datas of schizophrenia patients and healthy controls.

	**Patients (436)**	**Normal controls (291)**	***t*, or *x*^**2**^**	***p*-value**
Age (years)	45.99 ± 12.52	44.72 ± 9.03	1.578	0.115
**Sex**				
Male (%)	289 (66.28)	203 (69.76)		
Female (%)	147 (33.72)	88 (30.24)	0.963	0.326
HR (beats/min)	77.28 ± 9.84	74.05 ± 10.69	3.695	0.000^*^
QTc	417.94 ± 22.54	406.79 ± 19.61	7.071	0.000^*^
Male	415.74 ± 20.72	405.34 ± 19.67	5.647	0.000^*^
Female	422.25 ± 25.26	410.13 ± 19.51	4.117	0.000^*^

*HR, heart rate; QTc, corrected QT interval*.

* *Compared with the normal controls, p < 0.05*.

We used this normal control group to establish the upper limits of the normal values for measurement of QTc interval. According to the definition of QTc lengthening by Warner et al. (30) and Reilly et al. (16), the threshold of QTc interval prolongation was defined as two standard deviations (SD) above the mean value of the healthy controls. And in our present study the threshold of QTc interval prolongation was 444.7 ms for males and 449.2 ms for females.

### Demographic Data, Clinical Characteristics and Antipsychotic Treatment of Patients

The demographic and clinical variables of the subjects are shown in Table 2.

**Table 2:** Characteristics of schizophrenia patients with or without QTc prolongation.

**Characteristics**	**All patients**	**Patients without QTP**	**Patients with QTP**	***t*, *z* or *x*^**2**^**	***p-*value**
*N*	436	400	36		
Age (years)	45.99 ± 12.52	45.88 ± 12.64	47.11 ± 11.18	−0.563	0.574
**Sex**					
Male (%)	289 (66.28%)	271 (67.75%)	18 (50%)	4.656	0.031^*^
Female (%)	147 (33.72%)	129 (32.25%)	18 (50%)		
BMI	24.83 ± 4.43	24.84 ± 4.37	24.75 ± 5.14	0.109	0.913
Duration of illness (years)	20.65 ± 11.75	20.65 ± 11.75	20.74 ± 11.89	−0.173	0.863
Number of hospitalizations	3 (2, 6)	3 (2, 5)	5 (2, 9)	−1.795	0.073
Family history of mental illness (%)	67 (15.37%)	57 (14.25%)	10 (27.78%)	4.647	0.031^*^
**Antipsychotic**					
Monotherapy (%)	230 (53.12%)	214 (53.90%)	16 (44.44%)	1.186	0.276
Antipsychotic polypharmacy (%)	203 (46.88%)	183 (46.10%)	20 (55.56%)		
Antipsychotic dose (mg/day) (chlorpromazine equivalents)	250.00 (150.00, 450.00)	250.00 (146.25, 435.00)	240.00 (160.00, 515.75)	−0.370	0.711
Smoking behavior	170 (38.99%)	157 (39.25%)	13 (36.11%)	0.137	0.711
**PANSS**					
Positive subscore	8.88 ± 4.12	8.83 ± 4.12	9.39 ± 4.17	−1.012	0.312
Negative subsore	17.87 ± 6.83	17.80 ± 6.74	18.72 ± 7.80	−0.451	0.652
Concrete/Disorganized subscore	9.52 ± 3.32	9.41 ± 3.32	10.75 ± 3.21	–2.336	0.019^*^
Excited subscore	7.97 ± 3.23	7.89 ± 3.13	8.92 ± 4.17	−1.211	0.226
Depressive subscore	7.14 ± 2.57	7.13 ± 2.57	7.28 ± 2.58	−0.506	0.613
PANSS total score	77.87 ± 17.49	77.44 ± 17.36	82.61 ± 18.43	−1.704	0.089
HR (beats/min)	77.28 ± 9.84	77.16 ± 10.00	78.55 ± 7.97	−0.922	0.356
FPG (mmol/L)	5.08 ± 1.11	5.06 ± 1.10	5.28 ± 1.24	−1.603	0.109
CHOL (mmol/L)	4.24 ± 0.94	4.27 ± 0.93	3.99 ± 0.99	1.970	0.050
TG (mmol/L)	1.57 ± 1.04	1.57 ± 1.05	1.56 ± 0.96	0.177	0.860
HDL (mmol/L)	1.15 ± 0.32	1.15 ± 0.31	1.18 ± 0.39	−0.119	0.905
LDL (mmol/L)	2.59 ± 0.76	2.62 ± 0.76	2.27 ± 0.75	−2.676	0.007^*^
TP (mmol/L)	70.62 ± 5.03	70.77 ± 5.01	68.92 ± 5.05	2.121	0.035^*^
ALB (mmol/L)	42.14 ± 5.05	42.17 ± 5.14	41.83 ± 3.93	−0.637	0.524

*BMI, body mass index; PANSS, Positive and Negative Syndrome Scale; HR, heart rate; FPG, fasting plasma glucose; CHOL, cholesterol; TG, triglyceride; HDL, high density lipoprotein; LDL, low density lipoprotein; TP, total protein; ALB, albumin*.

* *p < 0.05*.

A total of 436 patients, including 289 males and 147 females, were included in this study. The average age of the patients was 45.99 (SD = 12.52), ranging from 18 to 70 years. The mean duration of illness was 20.65 years (SD = 11.75), ranging from 2 to 53 years.

Of the 436 patients, three patients' antipsychotic usage data were missing or unclear when collected from the medical records. The majority of the patients (230, 53.12%) were treated with single antipsychotic drug, while the other patients (203, 46.88%) were treated with two (197, 45.50%) or three antipsychotic drugs (6, 1.39%). Of the 230 patients treated with single antipsychotic drug, 38 were treated with olanzapine, 71 with risperidone, 88 with clozapine, 16 with aripiprazole, 8 with quetiapine, 3 with amisulpride, 1 with ziprasidone, and 5 with first-generation antipsychotics (4 with perphenazine and 1 with chlorpromazine). We converted the daily dose of antipsychotics to the equivalent dose of chlorpromazine using the standard guidelines (31), and the average dose was 327.22 ± 237.96 mg/ day.

### Comparison of Demographic and Clinical Characteristics Between Patients With QTc Prolongation and Non-QTc Prolongation

As presented in Table 2, QTc interval prolongation was present in 36 (8.26%) of 436 patients overall. More women (12.24%, 18 of 147) than men (6.23%, 18 of 289) displayed QTc interval prolongation (χ^2^ = 4.656, *p* = 0.031). The mean QTc interval was 415.74 ± 20.72 ms for males and 422.25 ± 25.26 ms for females, and there was significant difference between them (*t* = −2.871, *p* = 0.004).

Compared with patients without prolonged QTc, the patients with prolonged QTc interval had significantly lower TP levels (*t* = 2.121, *p* = 0.035), lower LDL cholesterol levels (*t* = −2.676, *p* = 0.007) and higher concrete/disorganized subscore (*t* = −2.336, *p* = 0.019). However, none of the significant differences passed the Bonferroni correction (α = 0.05/23 = 0.0022). Patients with prolonged QTc interval had higher PANSS scores (*t* = −1.704, *p* = 0.089), higher number of hospitalizations (*z* = −1.795, *p* = 0.073) and lower CHOL (*t* = 1.970, *p* = 0.050) than those without prolonged QTc interval, but the *p*-value was at the boundary level and did not reach a significant level.

Since age and sex were significant factors associated with QTc prolongation, we conducted an ANCOVA by using age and sex as covariates. After controlling the effect of age and sex, significant differences were also found in duration of illness (*F* = 106.705, *p* = 0.000), positive factor (*F* = 3.451, *p* = 0.017), FPG (*F* = 10.560, *p* = 0.000), CHOL (*F* = 2.905, *p* = 0.035), HDL cholesterol (*F* = 5.202, *p* = 0.002), LDL cholesterol (*F* = 3.837, *p* = 0.010), and ALB (*F* = 24.899, *p* = 0.000) between QTc prolongation and non- QTc prolongation groups. Moreover, the significant difference between QTc interval and duration of illness, FPG, HDL cholesterol passed the Bonferroni correction (α = 0.05/23 = 0.0022).

Furthermore, binary logistic regression analysis was performed to identify the risk factors for QTc prolongation, showing that the number of hospitalizations (OR = 1.042, *p* = 0.012), concrete/disorganized subscore (OR = 1.130, *p* = 0.019) and LDL cholesterol (OR = 0.549, *p* = 0.041) were associated with QTc prolongation. After controlling for the confounding factors, the number of hospitalizations (OR = 1.042, *p* = 0.012), concrete/disorganized subscore (OR = 1.126, *p* = 0.025) and LDL cholesterol (OR = 0.542, *p* = 0.038) were still associated with the prolongation of QTc interval. Within them, LDL seemed to be a protective factor for QTc prolongation. The results of binary logistic regression are shown in Table 3.

**Table 3:** Risk factors for QTc prolongation by binary logistic regression.

	**Crude odds ratio**	**95% C.I**.	***p-*value**	**Adjusted odds ratio from full model**	**95% C.I**.	***p-*value**
Female	1.542	0.718–3.316	0.267	1.497	0.693–3.236	0.305
Number of hospitalizations	1.042	1.009–1.076	0.012^*^	1.042	1.009–1.076	0.012^*^
Family history of mental Illness	1.862	0.764–4.537	0.171	1.891	0.771–4.638	0.164
Concrete/Disorganized subscore	1.130	1.020–1.252	0.019^*^	1.126	1.015–1.249	0.025^*^
LDL (mmol/L)	0.549	0.309–0.976	0.041^*^	0.542	0.304–0.966	0.038^*^
TP (mmol/L)	0.962	0.891–1.039	0.327	0.957	0.884–1.036	0.275

*LDL, low density lipoprotein; TP, total protein*.

* *p < 0.05*.

### Association Between QTc Interval and Clinical Variables in Patients

Pearson and Spearman correlation analysis indicated significant association between QTc interval and the following variables: sex, age, duration of illness, the number of hospitalizations, PANSS total score, FPG (all *p* < 0.05). And the significant association between QTc interval and age, duration of illness, PANSS total score passed the Bonferroni correction (α = 0.05/17 = 0.0029). The results of Pearson and Spearman correlation analysis are shown in Table 4. Furthermore, a multiple regression analysis showed that age (*t* = 2.687, *p* = 0.008), antipsychotic polypharmacy (*t* = 2.005, *p* = 0.046), PANSS total score (*t* = 2.325, *p* = 0.021), and LDL (*t* = −2.036, *p* = 0.043) were still associated with QTc interval. Among them, LDL was negatively correlated with QTc interval. The results of multiple regression analysis are shown in Table 5.

**Table 4:** Result of Pearson and Spearman correlation analysis on correlation of QTc.

	***r***	***p-*value**
Age	0.182	0.000^*^
Sex	0.137	0.004^*^
BMI	0.038	0.435
Duration of illness (years)	0.168	0.001^*^
Number of hospitalizations	0.126	0.011^*^
Family history of mental illness	0.077	0.107
Antipsychotic polypharmacy	0.076	0.115
Antipsychotic dose (mg/day) (chlorpromazine equivalents)	0.070	0.146
Smoking behavior	−0.086	0.073
PANSS total score	0.161	0.001^*^
FPG (mmol/L)	0.119	0.022^*^
CHOL (mmol/L)	−0.025	0.599
TG (mmol/L)	−0.003	0.948
HDL (mmol/L)	0.028	0.559
LDL (mmol/L)	−0.064	0.180
TP (mmol/L)	−0.030	0.532
ALB (mmol/L)	−0.089	0.066

*BMI, body mass index; PANSS, Positive and Negative Syndrome Scale; FPG, fasting plasma glucose; CHOL, cholesterol; TG, triglyceride; HDL, high density lipoprotein; LDL, low density lipoprotein; TP, total protein; ALB, albumin*.

* *p < 0.05*.

**Table 5:** The significant predictive variables associated with QTc by multiple regression analysis.

	**B**	**Std.Error**	**Beta**	***t***	***p-*value**
Female	4.286	2.960	0.092	1.448	0.149
Age	0.412	0.153	0.238	2.687	0.008^*^
Duration of illness (years)	0.052	0.159	0.027	0.328	0.743
Number of hospitalizations	0.279	0.147	0.105	1.900	0.058
Family history of mental illness	3.270	3.340	0.055	0.979	0.328
Antipsychotic polypharmacy	5.187	2.587	0.118	2.005	0.046^*^
Antipsychotic dose (mg/day) (chlorpromazine equivalents)	−0.002	0.006	−0.017	−0.275	0.783
Smoking behavior	−1.249	2.746	−0.028	−0.455	0.650
PANSS total score	0.162	0.069	0.128	2.325	0.021^*^
FPG (mmol/L)	0.644	1.081	0.034	0.596	0.552
LDL (mmol/L)	−3.220	1.581	−0.112	−2.036	0.043^*^
ALB (mmol/L)	0.366	0.282	0.082	1.296	0.196

*PANSS, Positive and Negative Syndrome Scale; FPG, fasting plasma glucose; LDL, low density lipoprotein; ALB, albumin*.

* *p < 0.05*.

## Discussion

We evaluated the prevalence, risk factors and clinical correlates of QTP in Chinese schizophrenia patients, which is not well-studied. Our study showed that the prevalence of QTP is 8.26% in Chinese patients with chronic schizophrenia. Up to now, there are few studies reported the prevalence of QTP in Chinese schizophrenia patients. And we found that the QTc interval of chronic schizophrenia patients was longer than that of normal controls, which is consistent with the most of previous studies (11, 16, 20). Furthermore, some demographic and clinical characteristics were found to be risk factors for QTP in these patients, including older age, higher number of hospitalizations, lower LDL, higher concrete/disorganized subscore, higher PANSS total score, or treatment with antipsychotic polypharmacy. Moreover, we found that LDL seemed to be a protective factor for QTP.

### The Prevalence of QTP

Our exploratory cross-section clinical study showed that the prevalence of QTP is 8.26% in hospitalized patients with chronic schizophrenia, which is in the middle of the range of prevalence (0.5–38%) reported by existing studies (11, 12, 15, 16, 20, 24). With respect to the prevalence of QTP in schizophrenia patients, up to now, there are few studies on Asian population, and even less on Chinese population. As far as we know, there is only one study reported the prevalence of QTc prolongation in schizophrenia patients in Chinese population and the reported prevalence is 4.5% (24).

In our present study the prevalence of QTP is 8.26% in hospitalized patients with chronic schizophrenia, which is relatively higher than that previous Chinese study and some foreign studies (12, 20, 24). The possible reasons may be that the patients in our study had a long duration of illness (20.65 ± 11.75 years) and a high frequency of antipsychotic polypharmacy (46.88%). Furthermore, QTP is also affected by genetic susceptibility, which is related to the race or ethnic origin. In addition, there is a limitation of the studies of QTP, which is the variation of cut-off values for abnormality, with a heterogeneity of ranging from 420 to 500 ms (11, 12, 15, 16, 20). That is to say, different researchers used different criteria for the definition of QTc prolongation. Therefore, due to the different criteria for the definition of QTc prolongation and the different characteristics of patients included, the comparison of the prevalence of QTP among different studies is limited.

### Gender Differences in the Prevalence of QTP

Our study showed gender differences in the prevalence of QTP in hospitalized patients with chronic schizophrenia, and the prevalence of QTP in females was higher than that in males (males: 6.23% vs. females: 12.24%). Moreover, female patients had longer QTc intervals than male patients. The results are consistent with most of the previous studies (11, 12, 15, 16, 32). However, there are also some studies showing that there is no gender difference in QTc interval prolongation, including a previous randomized multicenter trial (17) and a large epidemiological study (33). At present, many studies have found that female gender is an important risk factor for QTc interval prolongation (11, 12, 15, 16).

Up to now, the possible mechanisms of sex differences in QTc interval prolongation are still unclear. The sex differences in QTc interval do not exist at birth, but only appear at puberty (34–36). At the beginning of puberty the QTc interval of girls begin to lengthen compared with boys. As a result, the QTc interval of adult women is longer than that of men. This leads to the hypothesis that the sex differences in QTc interval is due to the sex hormone differences between men and women. Cardiomyocytes have a variety of sex hormone receptors, such as estrogen, progesterone and testosterone. Sex hormones could alter the electrical activity of cardiomyocytes through modulating ion channels. Although there are few literatures in this field, the fluctuation of sex hormone levels could lead to changes in the expression and behavior of myocardial ion channels (37). Although the hypothesis that estrogen may be responsible for sex differences in QTc interval prolongation has not been confirmed in humans yet, several clinical studies have shown that QTc interval may prolong in men with decreased testosterone (38, 39).

Also, in our study the results of correlation analysis showed that QTc interval was correlated with gender. However, it was not further confirmed by multiple regression. In other words, our study failed to detect that female gender is a significant risk factor of QTc interval prolongation. At this point, similar to our research, there are other studies that have failed to identify female gender as a risk factor for QTc interval prolongation, such as a Spanish registration study (40), a randomized, double-blinded, multicenter trial in Denmark (17) and a Chinese retrospective cohort study (25). The reasons for this situation may be the differences in sample size, characteristics of patients, and independent variables included in the regression equation.

### Effects of Age and Antipsychotic Polypharmacy on QTP

In our study, older age and treatment with antipsychotic polypharmacy were found to be risk factors for QTP in these patients. At present, it is generally believed that older age is a well-known risk factor for QTP (11, 12, 14). And our present research is in line with these studies. In addition, our study found that antipsychotic polypharmacy increased the risk of QTc interval prolongation. Several studies have shown that antipsychotic polypharmacy was associated with QTP (11, 12, 21). An Italian study of 725 patients treated with antipsychotics reported that the QTc interval of the patients receiving antipsychotic polypharmacy was longer than that of the patients receiving antipsychotic monotherapy and antipsychotic polypharmacy was associated with QTP (21). Another study of 2,411 schizophrenia patients reported that antipsychotic polypharmacy was positively associated with QTP (11). The possible explanation for this is that the risk of QTP is considered dose-dependent (14, 41, 42), which may also be related to the number of antipsychotic drugs taken by patients at the same time, as antipsychotic polypharmacy has consistently been found to be associated with increased equivalent antipsychotic dosage. Therefore, adding a second antipsychotic drug may further increase the risk of QTc interval prolongation as compared with monotherapy (42). However, a prospective, dual-center, naturalistic study of 65 patients with schizophrenia reported that there was no difference in the average QTc interval of patients receiving antipsychotic monotherapy and antipsychotic polypharmacy (15). The possible reason for this result may be that the sample size of this study was relatively small. A systematic review reported that antipsychotic polypharmacy was not definitely associated with QTc interval prolongation in three studies, one prospective study observed a significant QTc interval prolongation following antipsychotic polypharmacy, and the case reports showed that there was an increased risk of QTc interval prolongation in at least some patients who received antipsychotic polypharmacy (22). Therefore, the results of the existing studies in this area are inconsistent, and the relationship between antipsychotic polypharmacy and QTP deserves further investigation.

### The Relationship Between the Severity of Schizophrenia and QTP

For the first time, our study explored the relationship between PANSS scores and QTP, and found that concrete/disorganized subscore and PANSS total score were associated with QTP. And the more severe the schizophrenia is, the longer the QTc interval is. Up to now, there is no research to explore the relationship between QTP and the severity of schizophrenia. And we tried to analyze the possible reasons for the results we found. Usually, the more severe the patient's condition, the higher the dose of antipsychotic drugs will be used, which will aggravate the QTc prolongation. Moreover, with the development of pharmacogenetics, many genes related to QTP have been identified, such as KCNH2 (43, 44), CACNA1C (45), NOS1AP (44), KCNE1 (46) and so on. Among these genes, KCNH2 is frequently replicated for variations (14, 44, 47). In addition, several studies have identified that the KCNH2 gene was associated with the risk of schizophrenia (43, 48–50), including a case control study (43), a meta-analysis study of 1,158 schizophrenia patients and 1,704 controls (50). Then, some genes such as the KCNH2 gene may play an important role in both QTc interval prolongation and schizophrenia (50, 51). Therefore, we speculate that there may be some relationship between the QTc interval and the severity of schizophrenia. So far, there is no research on this aspect and we just did a preliminary study. And this field deserves our further study.

### The Relationship Between the Number of Hospitalizations and QTP

Furthermore, our study found that the high number of hospitalizations was also a risk factor for QTP. The possible reasons for the high number of hospitalizations may be that the patients are older and have a long duration of illness, then the number of hospitalizations will increase accordingly. It may also be due to the severity of schizophrenia, or poor treatment response and drug resistance, or poor medication compliance leading to recurrent episodes of schizophrenia. And these conditions have the possibility of increasing the number of hospitalizations and the probability of treatment with antipsychotic polypharmacy. As mentioned earlier in this article, the patient's old age, the severity of schizophrenia and antipsychotic polypharmacy were also associated with QTP.

### The Effect of LDL Cholesterol on QTP

We had an interesting finding in our present study. Compared with patients without prolonged QTc interval, the patients with prolonged QTc interval had significantly lower LDL cholesterol levels. After controlling the effect of age and sex, the significant difference still exists. Furthermore, both binary Logisitic regression analysis and multiple linear regression analysis showed that LDL cholesterol was negatively correlated with QTc interval prolongation. Maybe LDL cholesterol was a protective factor for QTP. This result was unexpected and may be contrary to what we thought. Up to now, there was no research has explored the relationship between LDL cholesterol and QTP. Moreover, there are few studies on the relationship between serum lipid levels and QTP. So far, there were only two studies have explored the relationship between serum lipid levels and QTc interval prolongation (12, 52). One was an Asian study of 2,553 schizophrenia patients and reported that hypercholesterolemia may be a risk factor for QTc interval prolongation in Asian patients with schizophrenia (52). However, another Spanish study of 171 Caucasian inpatients found that in patients aged ≥50 years, HDL cholesterol was associated with the prolongation of QTc interval (12). In the discussion of this article, the author found it difficult to explain this result (12). Moreover, some studies have shown that hypercholesterolemia has a protective effect on ventricular fibrillation in patients with myocardial infarction (53, 54). It could be seen that hypercholesterolemia is not always harmful to cardiac muscle cells. And their relationship may be much more complicated than we thought. In addition, as we all know, probucol is a cholesterol lowering drug and has the side effect of prolonging QTc interval (55, 56). And there was a Canadian study explored the mechanism of probucol inducing QTc interval prolongation (57). The study showed that probucol may induce QTc interval prolongation through decreasing the plasma-membrane hERG protein expression (57), as the decline of hERG channel is assumed to be a likely mechanism for the prolongation of QTc interval (44, 47). Furthermore, the Canadian study also found that the LDL cholesterol could efficiently impede the probucol effects by preventing probucol-induced reduction of the hERG current in hERG-expressing HEK cells (57). Similarly, there were many studies believed that the blockade of hERG channel was also a likely mechanism for QTP (22, 44, 47). Then, we could say that antipsychotics and probucol are both drugs that could prolong QTc interval by reducing the function of hERG. Therefore, we speculate that maybe LDL cholesterol could also protect QTc interval from prolongation in schizophrenia patients treated with antipsychotics just through such a similar mechanism as preventing probucol-induced reduction of the hERG current. However, there are few researches in this field at present, and the mechanism is very complex, which needs further study.

### Limitations

Several limitations of our present study should be noted here. First, our study is cross-sectional design, which makes it impossible to obtain the causal relationship between QTc interval prolongation and clinically relevant factors. Therefore, our findings seem to be exploratory, and we still could not draw definite conclusions. Second, our subjects were hospitalized Chinese patients with chronic schizophrenia who had been treated with antipsychotics for a long time. As a result, compared with the first-episode schizophrenia patients, the psychopathological symptoms of chronic patients may be more severe and the situation may be more complicated, which limited the generalization of this study. Third, compared with the first-episode schizophrenia patients, the chronic schizophrenia patients may have an increased risk of sedentary lifestyle, weight gain, obesity, type 2 diabetes, comorbid other diseases and concomitant medications. And this condition increases the potential risk factors for QTc interval prolongation in patients with chronic schizophrenia. Although we have included as many possible related factors in this study as possible compared with previous studies, there are still some factors that have not been included in this study, such as obesity, type 2 diabetes, comorbid other diseases, concomitant medications and so on. Fourth, although the patients enrolled in our study were hospitalized patients in the same hospital and the patients' diet and daily life were uniformly managed by the hospital, the heterogeneity of schizophrenia patients and antipsychotic drugs still exist. Therefore, our results in the study should be regarded as preliminary, which needs to be confirmed by further studies.

In conclusion, we found that the QTc interval of patients with chronic schizophrenia was longer than that of normal controls. The prevalence of QTP was 8.26% in our study. Furthermore, some demographic and clinical characteristics were found to be risk factors for QTP in these patients, including older age, higher number of hospitalizations, lower LDL cholesterol, higher concrete/disorganized subscore, higher PANSS total score, or treatment with antipsychotic polypharmacy. Moreover, LDL cholesterol seemed to be a protective factor for QTP.

## Data Availability Statement

The raw data supporting the conclusions of this article will be made available by the authors, without undue reservation.

## Ethics Statement

The studies involving human participants were reviewed and approved by the Human Ethics Committee of Tianjin Anding Hospital. The patients/participants provided their written informed consent to participate in this study.

## Author Contributions

HC, YZ, and TL: data curation, formal analysis, and writing—original draft. WY, SK, QJ, XJ, YuW, YaW, BY, CY, and ZW: data curation. YS: formal analysis. XZ: conceptualization and supervision. JL: conceptualization, writing—original draft, and supervision. All authors contributed to the article and approved the submitted version.

## Conflict of Interest

The authors declare that the research was conducted in the absence of any commercial or financial relationships that could be construed as a potential conflict of interest.

## Publisher's Note

All claims expressed in this article are solely those of the authors and do not necessarily represent those of their affiliated organizations, or those of the publisher, the editors and the reviewers. Any product that may be evaluated in this article, or claim that may be made by its manufacturer, is not guaranteed or endorsed by the publisher.
